# Sex-Specific Mechanisms of Perivascular Adipose Tissue (PVAT) Dysfunction in Prediabetes and Diabetes: From Metabolic Crosstalk to Vascular Outcomes

**DOI:** 10.3390/cells15181680

**Published:** 2026-09-17

**Authors:** Khadizatul Kobra, Md Arafat Hossain, Audreen Bandegan, Mohammad Moshiur Rahman, Atefeh Rabiee, Roshanak Rahimian

**Affiliations:** Department of Pharmaceutical Sciences, Thomas J. Long School of Pharmacy, University of the Pacific, Stockton, CA 95211, USA; k_kobra@u.pacific.edu (K.K.); m_hossain@u.pacific.edu (M.A.H.); a_bandegan@u.pacific.edu (A.B.); m_rahman4@u.pacific.edu (M.M.R.); arabiee@pacific.edu (A.R.)

**Keywords:** perivascular adipose tissue, prediabetes, diabetes, insulin resistance, vascular dysfunction, sex differences, adipokines, vascular tone

## Abstract

Perivascular adipose tissue (PVAT) is a metabolically active adipose depot surrounding most blood vessels and contributes to vascular homeostasis through local regulation of vascular tone, inflammation, and metabolic signaling. In prediabetes and diabetes, PVAT may shift from a predominantly vasoprotective phenotype toward a pro-inflammatory and pro-contractile state characterized by reduced release of protective mediators and increased cytokine, reactive oxygen species, and vasoconstrictor signaling. Vascular injury may, in turn, further alter the PVAT phenotype, creating bidirectional adipose-vascular crosstalk that may contribute to disease progression. Biological sex, reproductive aging, and sex-hormone signaling appear to influence PVAT morphology, cellular composition, secretory function, and vascular effects, but the strength of this evidence varies substantially across species, vascular beds, and disease models. Unlike recent broad reviews of PVAT biology, this review examines PVAT across the prediabetes-to-diabetes continuum while recognizing that direct longitudinal evidence tracking PVAT between these metabolic states remains scarce. We critically evaluate the direct, indirect, and still-limited evidence for sex-specific PVAT mechanisms and integrate findings on immune-cell activity, adipokine and redox signaling, and vascular function across prediabetes, established diabetes, and related metabolic conditions. Defining these mechanisms may support the development of appropriately tested, sex-informed strategies for preventing and treating diabetes-associated vascular complications.

## 1. Introduction

Diabetes, a major and rapidly growing cause of morbidity and mortality worldwide, is characterized by impaired glucose homeostasis resulting from insufficient insulin secretion, impaired insulin action, or both. The International Diabetes Federation (IDF) estimates that 589 million adults aged 20–79 years were living with diabetes in 2024 (approximately 1 in 9), with the number projected to reach 853 million by 2050 [1]. In the United States, current Centers for Disease Control and Prevention (CDC) estimates indicate that approximately 40.1 million people have diabetes and 115.2 million adults have prediabetes.

Prediabetes is an intermediate dysglycemic state that may revert to normoglycemia, persist, or progress to diabetes [2]. Many vascular changes begin at this stage before the onset of overt diabetes, such as endothelial dysfunction, reduced nitric oxide (NO) bioavailability, increased arterial stiffness, and altered vascular reactivity [3,4]. These early changes suggest that vascular dysfunction develops progressively during metabolic deterioration rather than arising only after the clinical onset of type 2 diabetes mellitus (T2DM) [4]. Beyond hyperglycemia and insulin resistance (IR), overt diabetes is strongly associated with obesity, dyslipidemia, hypertension, chronic inflammation, and endothelial dysfunction. These complications not only increase morbidity and mortality but also impose a substantial socioeconomic burden. Therefore, understanding the mechanisms that link metabolic dysfunction with vascular diseases during both prediabetes and diabetes is vital for identifying early therapeutic targets and delaying disease progression.

Perivascular adipose tissue (PVAT), a hybrid fat depot surrounding blood vessels, directly communicates with the vascular wall. Under healthy conditions, PVAT supports active endocrine, paracrine, and vasoregulatory functions, in addition to mechanical protection [5]. Experimental evidence also indicates that PVAT can directly regulate insulin-stimulated microvascular perfusion and glucose uptake through adipomuscular arterioles [6]. However, metabolic disorders may disrupt PVAT function, and PVAT dysfunction may represent a key mechanistic link between vascular dysfunction and metabolic disease [6,7]. Proposed mechanisms include increased inflammatory cytokines, reactive oxygen species (ROS), vasoconstrictors, and pro-fibrotic signals, together with reduced protective adipokines such as adiponectin, which may impair NO bioavailability, enhance vascular contraction, and promote vascular remodeling [5]. However, a causal role for PVAT dysfunction in driving vascular deterioration during the progression from prediabetes to diabetes, particularly in humans, has not been established. Thus, PVAT dysfunction is best considered a proposed mechanistic contributor, linking systemic metabolic dysfunction with local vascular disease rather than an established causal driver.

PVAT structure and function may differ by biological sex, but these differences are also shaped by vascular bed, age, reproductive stage, menopausal status, hormone therapy, metabolic status, and disease duration [8,9,10,11]. Reported sex-dependent features include PVAT morphology, inflammatory and immune-cell profiles, adipokine release, and modulation of endothelial function and vascular tone [7,8,10,11,12]. Estrogen and androgen signaling, adipose distribution, mitochondrial function, immune-cell polarization, and redox responses are plausible contributors, although direct mechanistic evidence remains limited [7,9,10,11]. In humans, direct evidence for sex-specific PVAT mechanisms would require demonstrating differences between women and men in vascular responses versus without adjacent PVAT or comparing PVAT-derived mediators directly or PVAT–vascular interactions, ideally supported by functional or ex vivo mechanistic studies.

Men often develop T2DM at a lower body mass index (BMI), whereas women with diabetes have a higher relative vascular risk than women without diabetes [13,14,15]. Our previous study also demonstrated sex-dependent vascular dysfunction during progression from prediabetes to T2DM in the University of California, Davis-type 2 diabetes mellitus (UCD-T2DM) rat model, though the direct involvement of PVAT was not explored [3]. In prediabetes and diabetes, sex-related factors may influence whether PVAT retains anticontractile signaling or shifts toward inflammatory and pro-contractile activity. However, direct longitudinal studies tracking PVAT from prediabetes to overt diabetes, particularly using sex-stratified designs, remain scarce.

Although the central focus of this review is sex-specific PVAT dysfunction across the transition from prediabetes to overt T2DM, much of the available evidence derives from studies examining prediabetes, obesity, IR, and established T2DM as distinct metabolic states rather than directly assessing PVAT changes during disease progression. Evidence from obesity, hypertension, and atherosclerosis is considered where it provides mechanistic insight relevant to this transition. These conditions should not be viewed as sequential stages of diabetes progression. Rather, obesity and hypertension are common cardiometabolic comorbidities that may coexist with IR and modify PVAT phenotype, whereas atherosclerosis represents a distinct vascular disease that shares several PVAT-associated inflammatory, oxidative, and adipokine pathways. Focusing on biological sex as a key modifier of PVAT structure and function across the transition from prediabetes to overt T2DM, we critically integrate evidence linking biological sex and sex-hormone signaling to changes in PVAT phenotype, adipokine and inflammatory signaling, redox balance, and vascular function. To distinguish direct PVAT involvement from broader systemic associations, we prioritize studies assessing PVAT tissue, PVAT-derived mediators, or vascular responses to PVAT removal or manipulation. Evidence based on circulating adipokines, systemic metabolic measures, or non-PVAT adipose depots is considered indirect and is used primarily to provide mechanistic context rather than evidence of a PVAT-specific mechanism.

## 2. Structural, Anatomical, and Cellular Features of PVAT

PVAT is a distinct adipose depot located adjacent to the adventitia of many arteries, veins, arterioles, and skeletal muscle microvessels. Its presence, thickness, and phenotype vary by species and vascular bed; it is absent around capillaries and may be sparse, discontinuous, or anatomically specialized in pulmonary and cerebral circulations [7,16]. Blood vessels contain tunica intima, media, and adventitia, with elastic laminae separating layers in many arteries. Because PVAT directly contacts the adventitia without a consistent fascial barrier, it is sometimes described as a functional fourth vascular layer or “tunica adiposa’’ [17]. This anatomical continuity enables local exchange of mediators between PVAT and the vascular wall.

PVAT differs from conventional adipose depots in its cellular origin, morphology, and function (Table 1) [9,18]. White adipocytes are primarily specialized for energy storage, whereas brown adipocytes contain abundant mitochondria and uncoupling protein 1 (UCP1) and support thermogenesis. Beige adipocytes arise within white-adipose depots and acquire inducible thermogenic features; beige adipose tissue is therefore a phenotype, not a synonym for subcutaneous adipose tissue (SAT) [19]. PVAT cannot be classified globally as subcutaneous or visceral fat. Instead, individual depots display white-, brown-, or beige-like characteristics according to anatomical location and physiological state. Thoracic aortic PVAT is commonly brown-like in rodents, whereas abdominal aortic, mesenteric, and coronary PVAT more often exhibit white-adipose features [20]. This regional heterogeneity is central to interpreting PVAT structure and function.

**Figure 1:**
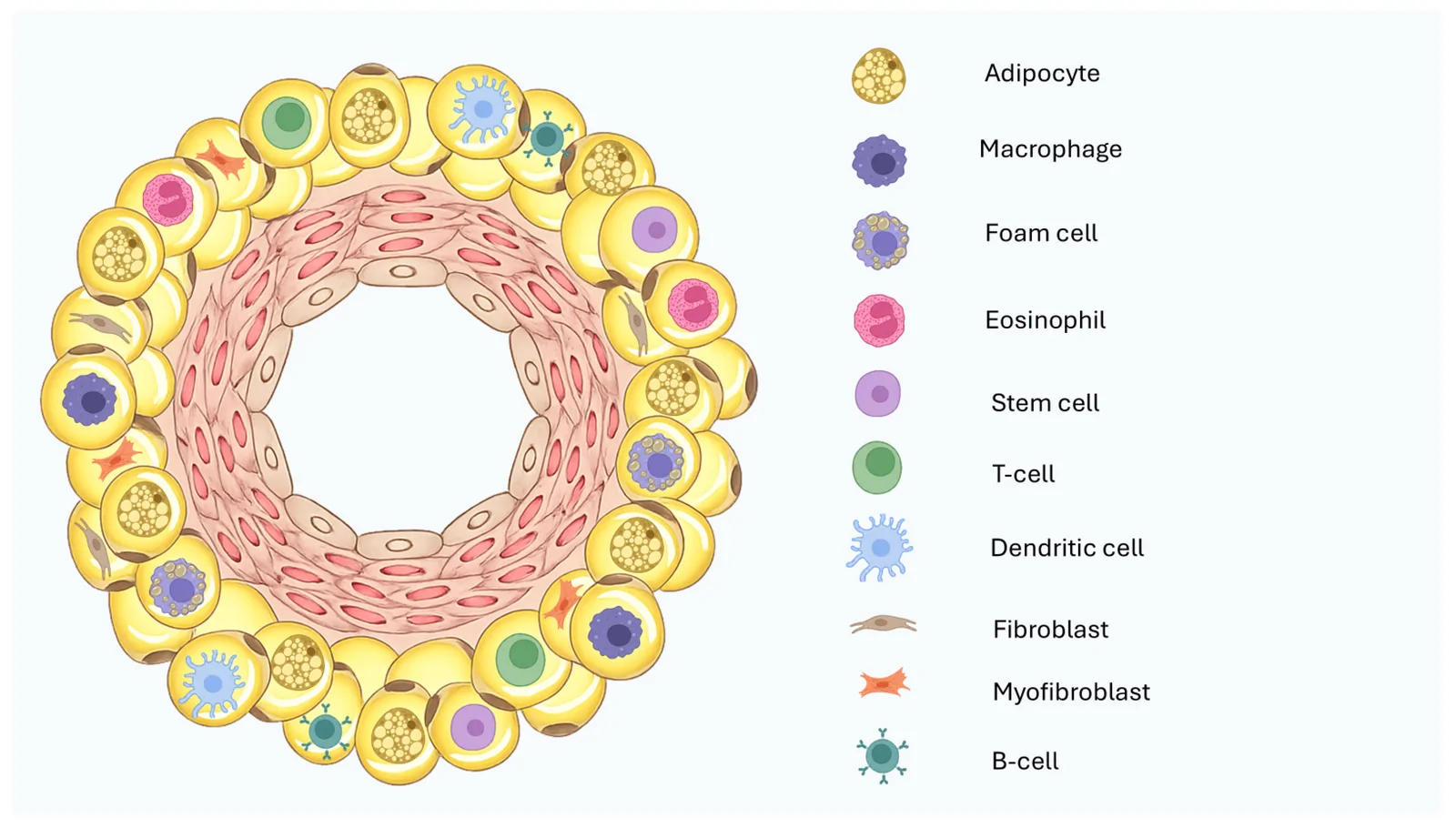
Cellular composition of perivascular adipose tissue (PVAT). Schematic cross-section of a blood vessel illustrating its organization from the lumen outward, including the endothelial cell layer and vascular smooth muscle cell layer, and surrounding PVAT. Adipocytes and resident stromal and immune cells contribute to physiological homeostasis, whereas foam cells and myofibroblasts are shown to represent cell states that become more prominent during pathological remodeling.

PVAT is a heterogeneous adipose depot containing mature adipocytes, adipocyte progenitors, endothelial and neural elements, fibroblasts, pericytes, and multiple immune-cell populations, including macrophages, lymphocytes, mast cells, and eosinophils (Figure 1) [5,21]. Reported cell proportions vary substantially with species, vascular bed, sex, disease state, tissue-dissociation method, gating strategy, and whether percentages are calculated among all cells or stromal-vascular cells. For example, studies of rat aortic and mesenteric PVAT (mPVAT)have reported sizable macrophage and lymphocyte populations, but these estimates should not be generalized to all healthy PVAT depots [9,20]. During metabolic and vascular disease, immune-cell recruitment, particularly macrophage accumulation, can shift the local secretory and redox environment toward inflammation [21].

Evidence for sex-related differences in PVAT is heterogeneous and should be interpreted by species and depot. Human imaging and tissue studies describe sex-associated differences in thoracic and pericoronary adipose phenotype but generally provide descriptive rather than causal evidence [8]. In rodents, aortic and mPVAT studies have reported sex differences in adipocyte size, progenitor markers, immune-cell composition, and inflammatory signaling [8,22]. Some findings suggest greater adipocyte hypertrophy or inflammatory signaling in male PVAT, whereas others identify depot-specific immune activation in females. These observations are not necessarily contradictory because they arise from different vascular beds, ages, and disease models. Therefore, mechanistic conclusions require direct manipulation of hormones, immune populations, or PVAT itself rather than comparison by sex alone.

## 3. Functions of PVAT

**Figure 2:**
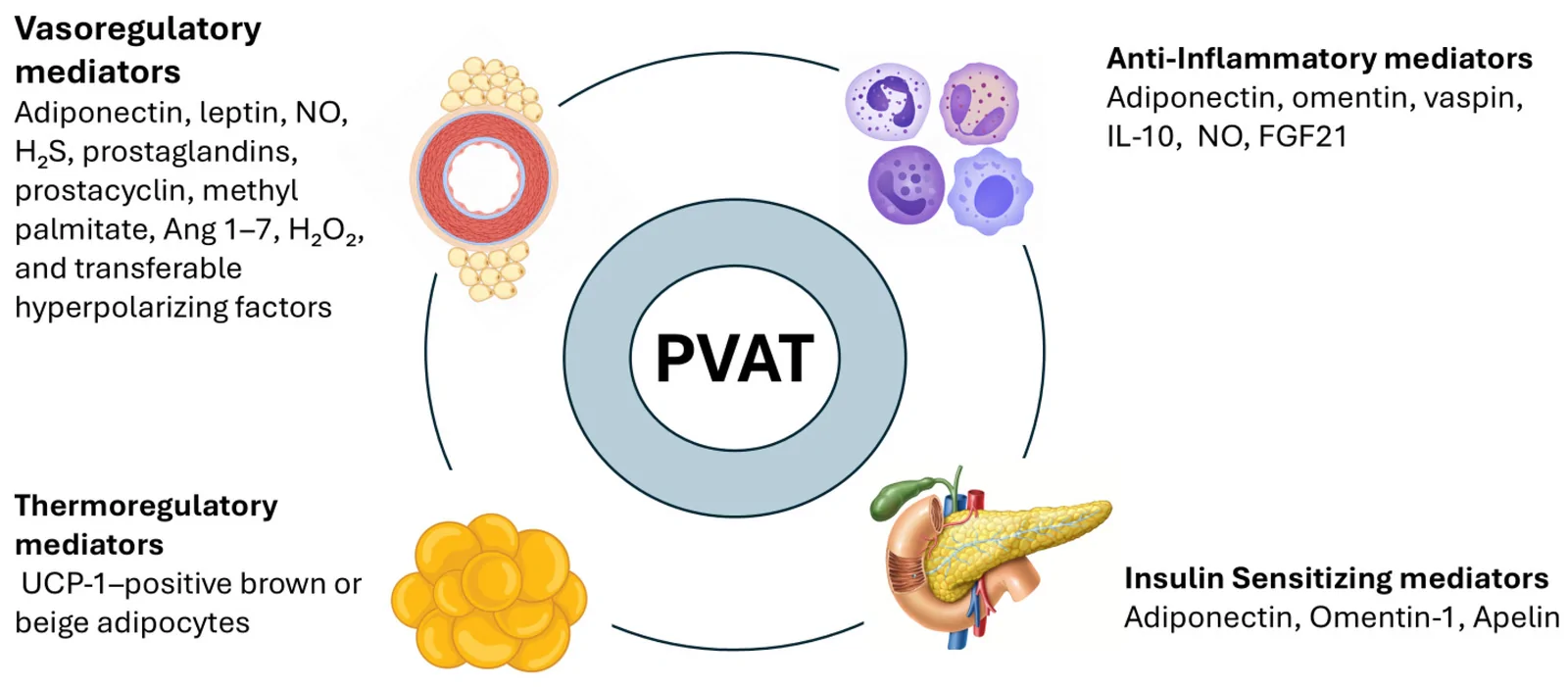
Functional roles of perivascular adipose tissue (PVAT) under healthy conditions. PVAT contributes to vascular homeostasis through the secretion of vasoregulatory, anti-inflammatory, thermogenic, and insulin-sensitizing mediators.

PVAT influences vascular biology through three connected communication routes. First, its adipose and stromal components provide mechanical support around the vessel. Second, endocrine release allows selected PVAT-derived mediators to enter the circulation and potentially affect distant tissues. Third, local paracrine and autocrine signaling permits direct exchange among PVAT adipocytes, immune and stromal cells, and the adventitia. PVAT-derived factors act on the vascular wall (outside-in), whereas endothelial and smooth-muscle signals can alter PVAT phenotype and secretory activity (inside-out) [23]. The physiological outcomes of vascular relaxation, immune restraint, thermogenic activity, and insulin-mediated microvascular recruitment share overlapping mediators and should therefore be considered as an integrated adipose-vascular network rather than isolated functions (Figure 2; Table 2).

### 3.1. Vasoregulatory Function

In 1991, Soltis and Cassis reported that intact PVAT reduced the contractile response of rat thoracic aorta to noradrenaline; removal of PVAT therefore increased agonist-induced contraction [24]. Subsequent animal and human studies have shown that PVAT causes relaxation by PVAT-derived relaxing factors (PVRFs) and contraction by PVAT-derived contracting factors (PVCFs), with the net effect depending on the vascular bed and physiological state [21]. Candidate relaxing mediators include adiponectin, leptin, NO, hydrogen sulfide (H_2_S), prostanoid, methyl palmitate, angiotensin 1-7 (Ang 1-7), hydrogen peroxide (H_2_O_2_), and transferable hyperpolarizing factors [25,26,27]. These factors act through endothelium-dependent and endothelium-independent mechanisms, including protein kinase G (PKG) signaling and activation of calcium-dependent potassium (K_Ca_) channels in vascular smooth muscle [21,27]. In metabolic disease, impaired NO signaling and increased inflammatory, oxidative, and contractile mediator release can shift this balance toward vasoconstriction.

**Table 2:** Selected PVAT mediators and context-dependent roles in physiological and pathological states.

Mediator	Major Source in PVAT	Role in Healthy PVAT	Role in Pathological PVAT	Vascular/Metabolic Outcome	References
Adiponectin	Adipocytes	Anti-inflammatory; activates AMPK/eNOS; enhances NO bioavailability and anticontractile activity	Reduced secretion ⟶ impaired AdiopR/AMPK/eNOS signaling ⟶ NO bioavailability	Endothelial dysfunction and impaired vascular relaxation	[28,29]
Omentin	PVAT adipocytes	Improves insulin sensitivity and suppresses inflammatory signaling	Reduced omentin ⟶ weaken insulin-sensitizing and anti-inflammatory signaling	Increased inflammation and endothelial dysfunction	[30,31]
Leptin	Adipocytes	Can support NO signaling and vascular regulation in some beds	Effects become pro-inflammatory and pro-oxidative with hyperleptinemia or leptin resistance	Oxidative stress and vascular injury	[32]
Apelin	Adipocytes/endothelium	Vasodilatory and insulin-sensitizing effects; direct PVAT evidence is limited	Dysregulation ⟶ altered endothelial -NO dependent signaling	Altered endothelial signaling and vascular function	[33]
Resistin	Adipocytes/macrophages	Physiological effects are context dependent	Increased resistin ⟶ inflammation and oxidative stress	Endothelial dysfunction and insulin resistance	[34]
Chemerin	Adipocytes	Modulates adipogenesis and immune cell recruitment	Increased chemerin ⟶ increased immune -cell recruitment and inflammatory signaling	Vascular remodeling and endothelial dysfunction	[35]
Interleukin-10 (IL-10)	Macrophages/immune cells	Anti-inflammatory; suppresses inflammatory cascades	Reduced IL-10 ⟶ weakens suppression of pro-inflammatory signaling	Loss of vascular protection	[36]
Transforming Growth Factor-β (TGF-β)	Adipocytes/immune cells	Tissue repair and matrix homeostasis	Excessive signaling ⟶ increased fibrosis	Vascular stiffening and remodeling	[37]
Tumor Necrosis Factor-α (TNF-α)	Macrophages/adipocytes	Low expression under physiological conditions	Increased TNF-α ⟶ increased inflammation	Insulin resistance and endothelial dysfunction	[38]
Interleukin-6 (IL-6)	Macrophages/adipocytes	Regulates immune homeostasis	Chronic elevation ⟶ sustained inflammatory signaling	Inflammation and impaired insulin signaling	[39]
Interleukin-1β (IL-1β)	Activated macrophages	Minimal under healthy conditions	Elevation ⟶ endothelial activation	Endothelial injury and inflammation	[40]
Monocyte chemoattractant protein-1(MCP-1)	Adipocytes/macrophages	Limited expression in healthy PVAT	Recruits monocytes/macrophages into PVAT	Chronic inflammation and immune cell infiltration	[41]
Interferon-γ (IFN-γ)	T lymphocytes	Minimal role in healthy PVAT	Increased IFN-γ ⟶ enhanced macrophage activation and inflammation	Macrophage activation and adipose dysfunction	[42]

### 3.2. Thermoregulatory Function

The thermogenic phenotype of PVAT varies substantially by species and anatomical location. In rodents, thoracic aortic PVAT displays prominent brown/beige adipose characteristics, and cold exposure can enhance thermogenic programs and modify vascular function, whereas abdominal aortic PVAT has a more WAT-like phenotype [9,43]. Functional evidence for PVAT thermogenesis is predominantly derived from rodent models and varies with vascular bed and experimental conditions. In mice, Chang et al. demonstrated that loss of periaortic PVAT following smooth muscle peroxisome proliferator-activated receptor gamma (PPARγ) deletion impaired intravascular thermoregulation and was associated with enhanced atherosclerosis, particularly under cold conditions [43]. These findings were obtained in a mouse model under specific cold-exposure conditions and therefore should not be generalized across PVAT depots or directly extrapolated to humans. Human PVAT exhibits substantial anatomical heterogeneity, and the contribution of thermogenic remodeling to vascular regulation across different vascular beds remains incompletely defined.

### 3.3. Anti-Inflammatory Function

The anti-inflammatory activity attributed to healthy PVAT reflects coordinated redox, adipokine, and immune-cell pathways rather than the action of a single mediator. PVAT-derived NO and adiponectin can support endothelial signaling, limit leukocyte adhesion, and restrain Nuclear Factor-κB (NF-κB) dependent inflammation [44,45]. Adiponectin may also favor anti-inflammatory macrophage polarization and autophagy [46]. Omentin-1, fibroblast growth factor (FGF21), vaspin, Interleukin-10 (IL-10), and UCP1-associated thermogenic pathways have been linked to reduced oxidative stress or inflammasome signaling, although much of this evidence derives from circulating adipokines, non-PVAT adipose tissue, or experimental models rather than direct vessel-specific PVAT measurements [47,48,49]. Accordingly, these mediators are best viewed as interacting candidate pathways whose relative importance varies by depot and disease context.

### 3.4. Insulin Sensitivity Function

**Figure 3:**
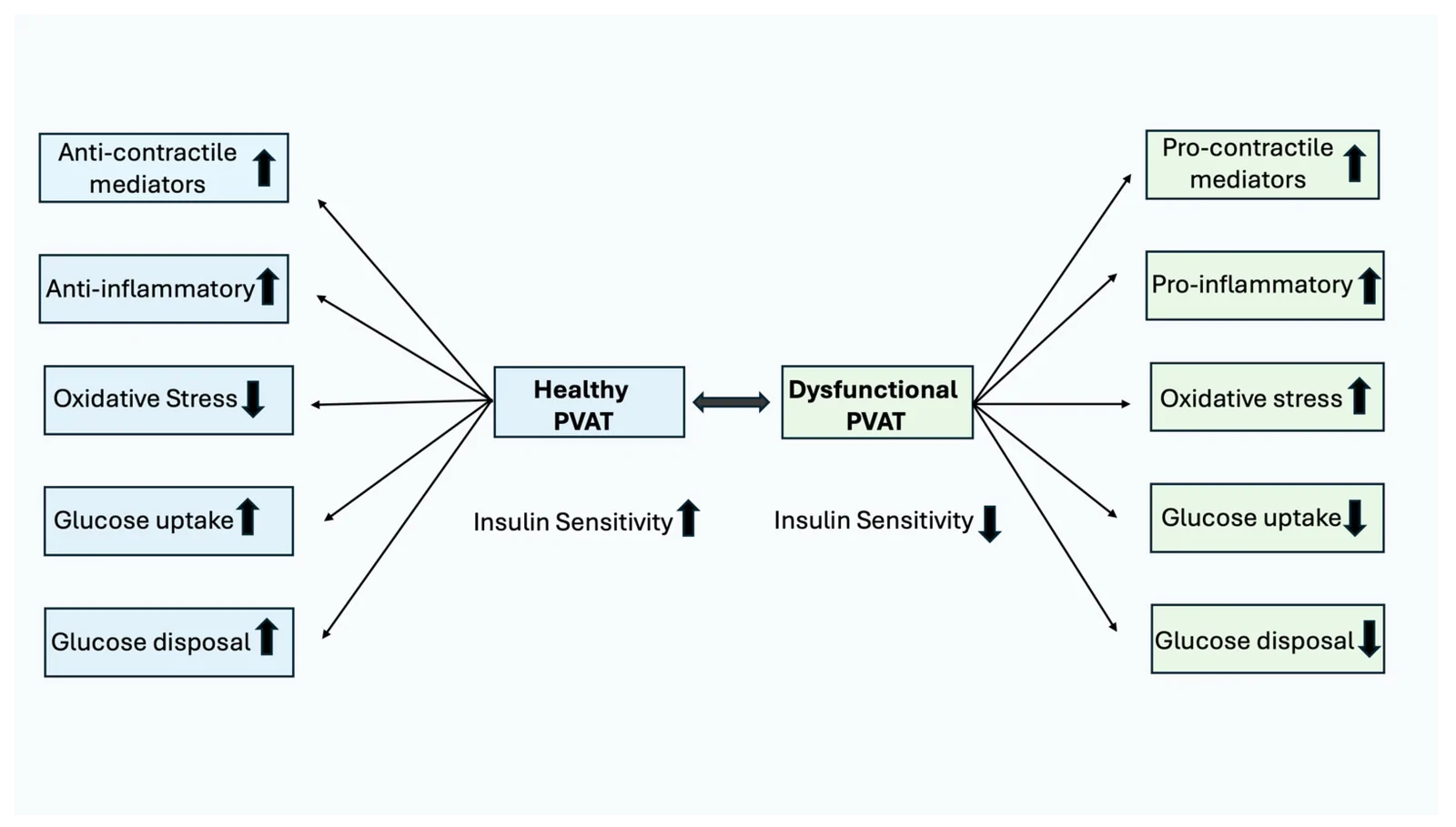
Functional characteristics of healthy and dysfunctional perivascular adipose tissue (PVAT) in the regulation of insulin sensitivity. Healthy PVAT is associated with greater anti-contractile and anti-inflammatory signaling, lower oxidative stress, and increased insulin sensitivity, glucose uptake, and glucose disposal. Dysfunctional PVAT shows the opposite pattern. Arrows indicate the direction of change relative to the contrasting PVAT phenotype. Upward (↑) and downward (↓) arrows indicate increased and decreased conditions, respectively, and the bidirectional (↔) arrow indicates reversible conditions.

One important metabolic function of PVAT is to modulate insulin sensitivity. Under physiological conditions, PVAT enhances insulin action primarily by modulating endothelial function and microvascular perfusion, key determinants of glucose delivery to insulin-sensitive tissues. A study in male mice demonstrated that surgical removal of PVAT abolished insulin-induced increases in skeletal muscle perfusion and glucose uptake, supporting a direct PVAT-specific mechanism rather than solely systemic insulin action [6,50]. This represents a local PVAT–vascular interaction and should be distinguished from insulin’s broader systemic metabolic actions. These functions are carried out through the coordination of vascular and metabolic signaling (Figure 3). Adiponectin is an important PVAT-derived adipokine that links vascular perfusion with systemic metabolic regulation. Adiponectin R1-adenosine monophosphate-activated protein kinase (AdipoR1–AMPK) signaling promotes fatty-acid oxidation and can improve insulin action, whereas PVAT surrounding skeletal-muscle microvessels contributes to insulin-induced vasodilation and glucose delivery [28,51,52]. Higher circulating adiponectin has been associated with lower risk of prediabetes or T2DM in several populations, but circulating concentrations do not identify PVAT as the source [29,53]. Thus, adiponectin is a plausible mediator of the prediabetes-to-diabetes transition, although direct longitudinal evidence from vessel-specific PVAT remains limited.

Omentin-1 has insulin-sensitizing, anti-inflammatory, and endothelial effects, and some studies have directly examined its action in PVAT-containing vessels [30,31,47]. By contrast, much of the apelin literature concerns circulating apelin or skeletal-muscle and systemic metabolic signaling. Apelin can activate phosphoinositide 3-kinase (PI3K)/Akt and extracellular signal-regulated kinase (ERK) pathways and enhance glucose uptake, but direct evidence that PVAT-derived apelin controls vascular function during prediabetes remains limited [54,55]. IL-10 and transforming growth factor beta (TGF-β) may also contribute to local immune regulation, although their effects are context- and concentration-dependent [54,56].

Biological sex can influence PVAT function, although the direction and magnitude of sex-related differences vary by vascular bed and disease context. Importantly, the core PVAT functions are not uniform between sexes. Rather, the contributions of PVAT-derived mediators, adipokines, redox signaling, and immune signaling differ between males and females. Section 6 discusses how this dimorphism affects the development of cardiometabolic diseases in males and females, as well as their responses to treatment.

## 4. PVAT Involvement in Disease Pathology

In certain pathological conditions, such as IR, prediabetes, diabetes, obesity, hypertension, and atherosclerosis, PVAT undergoes functional and phenotypic alterations. PVAT also undergoes secretory changes, releasing several PVCFs. Thus, PVAT can lose its protective anti-inflammatory and anticontractile properties and shift toward a pro-contractile, pro-inflammatory phenotype, contributing to vascular dysfunction. The relationship between PVAT dysfunction and metabolic diseases is bidirectional. The following subsections summarize how different disease states alter PVAT biology and how dysfunctional PVAT further contributes to disease progression and vascular impairment.

### 4.1. Prediabetes and Diabetes

IR, prediabetes, and diabetes form a metabolic continuum, but the timing and direction of PVAT changes across this continuum remain incompletely defined. Evidence derived predominantly from animal models indicates that early metabolic stress can promote adipocyte hypertrophy, mitochondrial dysfunction, altered adipokine secretion, and immune-cell recruitment within PVAT [23]. Although human studies support an association between systemic inflammation and metabolic dysfunction during prediabetes, direct longitudinal evidence demonstrating PVAT involvement in the progression from prediabetes to T2DM remains limited [57]. Lower circulating adiponectin is associated with insulin resistance and progression toward T2DM, whereas experimental restoration of adiponectin can improve selected metabolic outcomes [51,58,59]. PVAT studies further link altered adiponectin signaling to vascular tone and insulin-mediated perfusion. In skeletal-muscle resistance arteries, PVAT from control mice promoted insulin-induced vasodilation through adiponectin-AMP-activated protein kinase alpha 2 catalytic subunit (AMPKα2) signaling, whereas PVAT from obese diabetic db/db mice secreted less adiponectin and failed to support this response; adiponectin blockade abolished the protective effect of control PVAT [60]. Additional mechanistic studies in non-diabetic models demonstrated adiponectin-dependent PVAT anticontractile effects through large-conductance calcium-activated potassium (BK_Ca_) channels and a role for PVAT-derived adiponectin in thoracic aortic relaxation [61,62]. As hyperglycemia and lipotoxicity become established, oxidative and inflammatory signaling may further suppress protective adipokines and impair PVAT-mediated vascular regulation [63]. However, these studies do not establish longitudinal changes in PVAT-derived adiponectin from prediabetes to overt diabetes. Other PVAT-associated mediators, including omentin-1, leptin, apelin, and resistin, may contribute, but their causal roles and depot specificity require direct testing.

Circulating omentin-1 concentrations are often reduced in impaired glucose tolerance and T2DM and correlate inversely with IR and metabolic-syndrome severity [30,31]. These systemic observations are consistent with, but do not prove, reduced secretion from PVAT. Evidence comes from diabetic Goto-Kakizaki rats, in which omentin-1 treatment partially restored the anticontractile function of thoracic aortic PVAT and reduced PVAT inflammatory and oxidative markers [64]. However, this demonstrates a functional effect of exogenous omentin-1 on diabetic PVAT rather than reduced endogenous PVAT-derived omentin-1 secretion. By contrast, PVAT produces leptin locally, which can influence vascular smooth muscle and endothelial signaling through Janus Kinase 2 (JAK2)/STAT3, PI3K/Akt, and mitogen-activated protein kinase (MAPK) pathways [65]. In obesity and diabetes, hyperleptinemia and leptin resistance may favor inflammatory cytokine production, ROS generation, and impaired endothelium-dependent vasodilation [66,67,68].

PVAT-derived resistin may contribute to the link between metabolic dysfunction and vascular injury, although its role across the transition from prediabetes to T2DM remains insufficiently defined [34]. Furthermore, experimental and clinical studies have demonstrated elevated apelin levels in obesity and insulin-resistant states, and apelin levels have been positively correlated with hyperinsulinemia [54,55]. Thus, understanding the mechanisms underlying PVAT dysfunction in prediabetes and diabetes is essential for identifying early therapeutic targets to restore vascular health and improve metabolic outcomes. The complex interplay between PVAT-derived mediators, vascular function, and insulin signaling has emerged as a critical area of investigation in cardiometabolic research.

### 4.2. Other Vascular Diseases and Metabolic Complications

#### 4.2.1. Obesity

Obesity is one of the major metabolic conditions associated with PVAT remodeling and vascular dysfunction. Excess adipose tissue accumulation promotes oxidative stress, chronic inflammation, tissue hypoxia, and immune cell infiltration within adipose depots, ultimately disrupting the protective anticontractile properties of PVAT [54,69]. A recent study further suggests that obesity induces “whitening” of PVAT, characterized by reduced UCP1 expression, mitochondrial dysfunction, and increased lipid accumulation [43,70]. In obesity and metabolic disease, the thermogenic phenotype of PVAT becomes impaired, reducing energy expenditure and decreasing the release of vasodilatory mediators such as NO, adiponectin, and H_2_S [43,71,72]. Experimental studies in high-fat-diet (HFD) animal models have demonstrated reduced H_2_S production in the PVAT, leading to diminished vasorelaxation and endothelial dysfunction [73,74]. Additionally, chronic hyperleptinemia and leptin resistance observed in obesity may contribute to endothelial dysfunction, sympathetic nervous system activation, vascular remodeling, and hypertension [75,76].

Experimental studies in obese and diabetic animal models have demonstrated increased macrophage infiltration and elevated expression of inflammatory adipokines in the PVAT, specifically resistin [77,78]. Secreted by macrophages, resistin is upregulated by inflammatory cytokines such as TNF-α and IL-6 and has been implicated in IR and endothelial dysfunction [77]. Mechanistically, resistin activates Toll-like receptor 4 (TLR4)- and cyclase-associated protein 1 (CAP1)-mediated signaling pathways to enhance cytokine production and reduce NO bioavailability [79]. In vascular smooth muscle cells (VSMCs), resistin has also been shown to stimulate osteopontin (OPN) production through activator protein 1 (AP-1) signaling, contributing to vascular inflammation and remodeling [78,80]. These findings support the concept that inflammatory PVAT signaling plays an active role in obesity-associated vascular disease and warrants further exploration.

#### 4.2.2. Hypertension

Dysfunctional PVAT may contribute to the development and progression of hypertension by altering vascular tone and promoting inflammatory and oxidative signaling. Animal models, including spontaneously hypertensive rats (SHR), Ang II-infused mice, and deoxycorticosterone acetate (DOCA)-salt hypertension, frequently show loss of the PVAT anti-contractile effect, immune-cell infiltration, increased local renin–angiotensin–aldosterone system (RAAS) activity, and oxidative stress [16,81,82,83]. These changes are often accompanied by reduced adiponectin and activation of the angiotensin II type 1 (AT1) receptor, nicotinamide adenine dinucleotide phosphate (NADPH)-oxidase (NOX), NLRP3-inflammasome, and mitochondrial pathways [84]. The relative contribution of PVAT compared with renal, neural, endothelial, and systemic mechanisms nevertheless remains model-dependent.

Intriguingly, interventions targeting RAAS signaling, oxidative stress, or inflammatory pathways have been shown to partially restore PVAT function and improve endothelial-dependent vasodilation, highlighting PVAT as a potential therapeutic target in hypertension [85]. Studies in prediabetic hereditary hypertriglyceridemic rats suggest that PVAT may initially play a compensatory role during the early stages of hypertension [73]. In these models, H_2_S produced within the arterial wall exhibited pro-contractile effects, whereas PVAT generated stronger anti-contractile signaling to compensate for increased vasoconstriction [73]. Thus, PVAT dysfunction may contribute to the pathogenesis of hypertension-associated vascular dysfunction. However, its contribution relative to systemic blood-pressure regulatory mechanisms remains context-dependent.

#### 4.2.3. Atherosclerosis

Dysfunctional PVAT can promote atherogenesis through local inflammatory and “outside-to-inside” signaling. In Apolipoprotein E (ApoE)^−/−^ mouse models, PVAT exhibits increased cytokine and chemokine expression, macrophage and T-cell infiltration, and reduced protective B-1-cell populations [71,86]. Experimental transplantation or restoration of adiponectin signaling can attenuate plaque formation, supporting a causal contribution in selected models [87]. Human studies provide evidence of inflammatory remodeling of coronary PVAT: pericoronary adipose tissue from patients with coronary artery disease shows activation of inflammation- and atherosclerosis-related pathways, while CT- and PET-based imaging demonstrates increased pericoronary inflammatory activity [88,89,90]. However, these observational findings cannot establish a causal role for PVAT in atherosclerosis. Consistent with a potential mechanistic role, PVAT-conditioned medium from HFD Dahl S rats enhanced inflammatory cytokine secretion by activated T cells [91]. Collectively, these findings establish dysfunctional PVAT as an active participant in atherogenesis and suggest that restoration of PVAT homeostasis may have therapeutic potential.

In general, biological sex may influence PVAT-vascular crosstalk significantly and should be taken into consideration to understand disease pathology and precise treatment. How sex hormones can regulate PVAT function is discussed in the subsequent sections.

## 5. Sex Hormonal Control of PVAT Responses

Sex differences in PVAT-mediated vascular function may be influenced by sex hormones; however, evidence of sexual dimorphism alone does not establish direct hormonal or receptor-mediated regulation. Throughout this section, we distinguish evidence demonstrating a sex difference from evidence directly establishing the involvement of a specific sex hormone or hormone receptor in PVAT-mediated vascular function. Among the major sex hormones, estrogen currently has the strongest experimental support for involvement in PVAT-mediated vascular protection. Estrogen has well-established vasodilatory, anti-inflammatory, and metabolic actions, including activation of endothelial nitric oxide synthase (eNOS) and enhancement of NO bioavailability [92].

Recent experimental evidence provides more direct support for estrogen receptor α (ERα)-mediated regulation of PVAT function. In female mice, adipose-specific ERα overexpression enhanced the anticontractile effect of mPVAT and protected against HFD-induced PVAT dysfunction through NOX4-derived H_2_O_2_ signaling, while pharmacological NOX4 inhibition abolished the enhanced response. Complementary evidence for estrogen-dependent PVAT regulation comes from ovariectomized hypertensive rats, in which estrogen replacement restored PVAT-mediated anticontractile function through NOX4-dependent H_2_O_2_ signaling [92].

Estrogen may additionally influence PVAT through broader effects on adipose distribution, adipokines, energy sensing, and tissue hypoxia [93]. Sex differences have been reported in circulating adipokines, including adiponectin, while altered circulating omentin-1 and adiponectin concentrations have also been observed in T2DM; however, these systemic measurements should not be interpreted as direct evidence of PVAT secretion or estrogen-dependent PVAT regulation [94,95]. Experimental studies examining vascular function in association with PVAT also provide evidence for sex-dependent PVAT phenotypes. Female prediabetic rats showed better-preserved endothelial function and PVAT-associated compensatory vasoactive signaling than males [73]. Importantly, these findings demonstrate a sex-dependent phenotype but do not by themselves identify estrogen as its cause. Estrogen supplementation in ovariectomized female rats also reduced periaortic adipose tissue hypoxia, accompanied by decreased hypoxia-inducible factor-1 alpha (HIF-1α) and vascular endothelial growth factor (VEGF) expression [93]. Separately, pharmacological activation of AMPK in PVAT reduced inflammatory signaling and improved acetylcholine (ACh)-dependent vasodilation and endothelial eNOS phosphorylation, supporting AMPK/eNOS signaling as a relevant PVAT pathway [93,96]. However, because this study did not directly examine estrogen signaling, it does not establish AMPK/eNOS as an estrogen-dependent PVAT mechanism.

Evidence from non-PVAT adipose tissue further suggests receptor-specific effects. Adipocyte ERα deficiency promotes adipose inflammation and fibrosis, whereas estrogen receptor β (ERβ) may exert compensatory metabolic effects when ERα signaling is deficient [97]. Increased adipose ERβ expression in aged female rats has also been associated with increased intracellular but reduced circulating adiponectin [98]. However, because these ERβ findings derive from non-PVAT adipose tissue, the role of ERβ in PVAT-mediated vascular regulation remains unresolved [97,98].

In contrast, while evidence for estrogen-related regulation of PVAT is stronger, androgen involvement in PVAT-mediated vascular function remains less well defined. Androgen receptor signaling influences adipocyte differentiation, fat distribution, lipid metabolism, insulin sensitivity, and inflammatory pathways, but most of this evidence derives from non-PVAT adipose tissue [99,100,101,102]. Testosterone also exerts direct vascular effects that vary with experimental context: acute exposure can induce vasorelaxation, whereas prolonged exposure has been associated with vascular inflammation, oxidative stress, and endothelial dysfunction in selected models [11,99,103,104]. These studies establish that testosterone can influence vascular function but do not determine whether PVAT participates in these responses.

Direct evidence for progesterone-dependent regulation of PVAT-mediated vascular function is currently sparse. Although progesterone has been linked to endothelial responses and pregnancy-associated changes in adipokine expression within uterine-artery-associated PVAT, these findings do not establish a direct progesterone–PVAT–vascular mechanism [100,101]. Thus, current evidence is insufficient to establish progesterone as a regulator of PVAT-mediated vascular function. Progesterone therefore represents a priority knowledge gap, particularly regarding receptor-specific actions in PVAT and its potential role during pregnancy and the menopausal transition.

In conclusion, although sex hormones are plausible regulators of PVAT phenotype, evidence for specific hormone-dependent PVAT mechanisms remains uneven. Establishing causality requires manipulating hormone availability or receptor signaling and assessing PVAT-dependent vascular function. Findings based solely on circulating hormone levels, receptor expression, or effects observed in other adipose depots or vascular tissues should therefore be considered supportive rather than definitive, particularly for progesterone, androgen signaling, and the relative contributions of ERα and ERβ.

## 6. Sex-Specific PVAT Involvement in Disease Pathology

There is an association between sex hormonal levels and PVAT involvement in a sex- and disease-state-dependent manner. As metabolic diseases progress, the sex-hormonal balance and PVAT function become highly dysregulated [102]. Likewise, in IR, prediabetes, and diabetes, ovarian and adrenal androgen overproduction leads to hyperandrogenism and relative estrogen deficiency in women [105,106]. In T2DM and obesity, testosterone levels decrease while aromatase activity increases and estradiol rises in males, disrupting the testosterone-to-estrogen ratio; this estrogen imbalance leads to PVAT dysregulation and disease exacerbation [107]. In this section, we will discuss how this sexual dimorphism of PVAT contributes to the development of prediabetes, diabetes, and other cardiometabolic diseases.

### 6.1. Sex-Specific PVAT Dysfunction in Prediabetes and Diabetes

Whether PVAT buffers or amplifies vascular stress during prediabetes appears to depend on sex and hormonal status, but direct evidence is limited. In a mild-hypercaloric rat model, intact females were relatively protected from metabolic and cardiovascular abnormalities and thoracic aortic PVAT dysfunction, whereas ovariectomy was associated with a worsened phenotype [108]. These findings implicate ovarian hormonal status in PVAT responses during prediabetes, but do not establish that PVAT dysfunction itself causes the associated vascular impairment. Moreover, sex differences may vary across vascular beds and disease stages. Accordingly, in our previous study using the UCD-T2DM model, mesenteric artery relaxation was more severely impaired in prediabetic males than in females, whereas this pattern reversed after progression to overt diabetes, with greater impairment in diabetic females [3]. However, because PVAT was not experimentally manipulated, these findings support sex-dependent vascular dysfunction but do not establish a specific role for PVAT [3].

**Figure 4:**
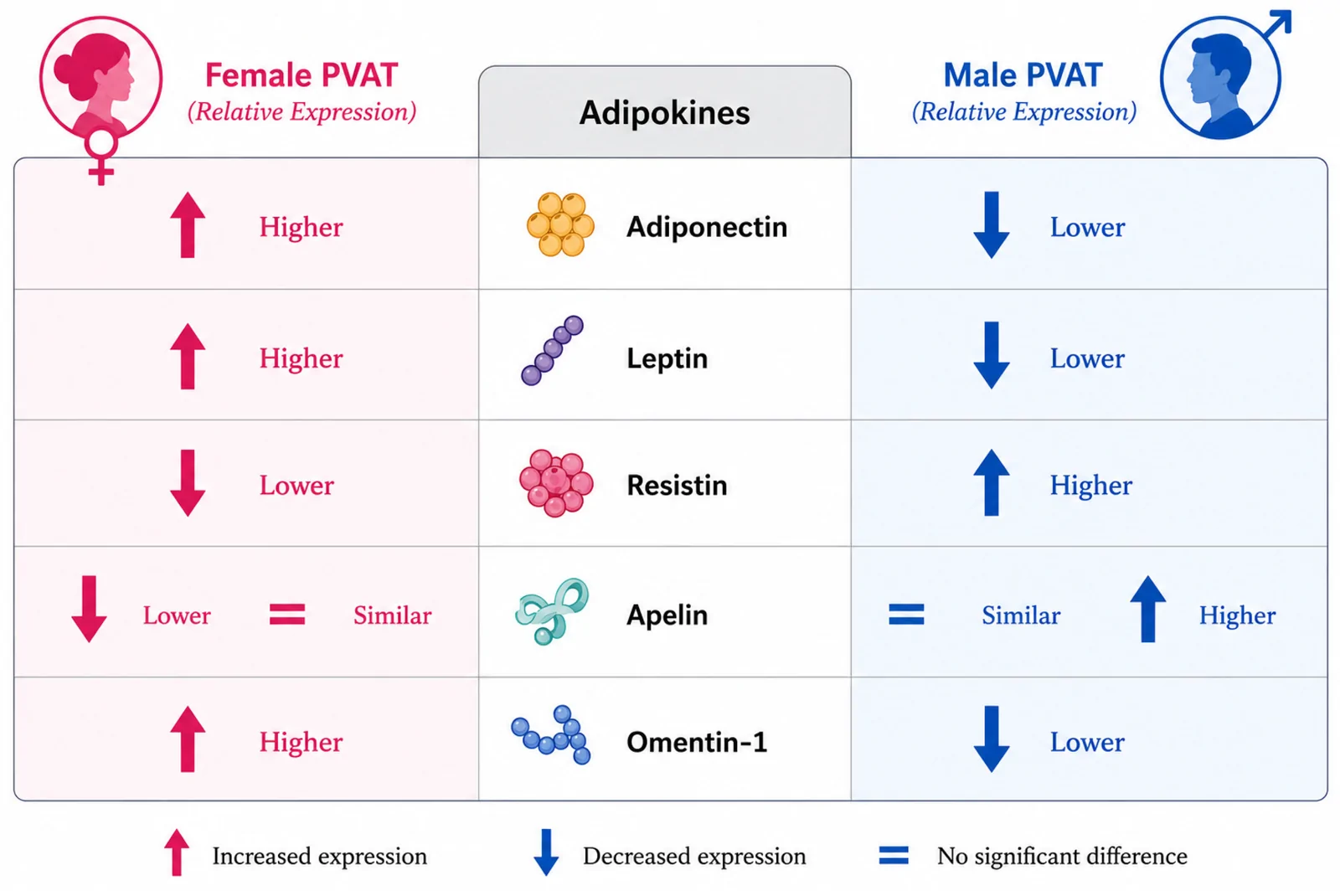
Sex-specific differences in perivascular adipose tissue (PVAT)-derived adipokine secretion. Female PVAT generally exhibits higher adiponectin, leptin, and omentin-1, lower resistin, and similar or lower apelin levels than male PVAT. In contrast, male PVAT shows lower adiponectin, leptin, and omentin-1, higher resistin, and similar or higher apelin levels. Upward (↑) and downward (↓) arrows indicate increased and decreased expression, respectively, while the equals sign (=) denotes no significant sex difference. Pink and blue columns represent females and males, respectively.

Adipokine expression levels also differ by biological sex and may contribute to metabolic and vascular dysfunction in prediabetes, diabetes, and IR (Figure 4). Sex-specific evidence directly from PVAT-derived adiponectin remains limited. In porcine coronary arteries, female PVAT produced greater adiponectin-dependent vasorelaxation than male PVAT despite similar adiponectin expression and release between sexes, suggesting sex differences in vascular responsiveness rather than adiponectin production [109]. Conversely, in patients undergoing coronary artery bypass grafting, Antonopoulos et al. directly assessed internal mammary artery PVAT and demonstrated altered adiponectin expression in T2DM; ex vivo artery–PVAT co-incubation further supported paracrine crosstalk between PVAT adiponectin and vascular redox signaling, but these findings do not establish a sex-specific effect [110]. In humans, lower circulating adiponectin is associated with progression toward T2DM and is generally lower in males than females [53,111,112]. Sex-specific adiponectin signaling has also been observed in human monocyte-derived macrophages. Males showed reduced AdipoR2–PPAR-α signaling and a heterogeneous inflammatory profile, whereas females demonstrated increased AdipoR1 expression without changes in AdipoR2–PPAR-α signaling [52]. However, these findings are not PVAT-specific, and circulating adiponectin differences may also reflect age, adiposity, body-fat distribution, and hormonal status. Although having the same body fat mass, females exhibit higher circulating adiponectin levels [113]. Because subcutaneous fat is more active than visceral fat in producing insulin-sensitizing adipokines, greater subcutaneous fat may also explain why women have higher adiponectin levels [114]. Experimental obesity studies have also shown greater inflammation and extracellular-matrix remodeling in male mPVAT than in female mPVAT; however, this sex-specific PVAT phenotype cannot be attributed directly to lower circulating adiponectin [115,116]. Collectively, direct PVAT findings together with supportive systemic data suggest a plausible sex-dependent role for adiponectin signaling in metabolic and vascular dysfunction, although direct evidence linking sex-specific PVAT-derived adiponectin to prediabetes or diabetes remains lacking.

The function and regulation of omentin-1 may also differ by sex, although direct PVAT-specific evidence is limited. In obese patients undergoing Roux-en-Y bariatric surgery, omentin-1 mRNA was directly measured in mPVAT and was higher in females without vascular/PVAT pathology than in males; however, expression was reduced in females with pathological vascular/PVAT remodeling [117]. Men had significantly lower fasting serum omentin levels than female subjects, and these levels were negatively correlated with IR (HOMA-IR) [94]. Since men tend to accumulate more visceral fat closely tied to cardiometabolic risks, lower omentin levels may increase vascular vulnerability [114]. In line with depot-specific regulations, omentin gene expression in visceral fat is significantly lower in women with morbid obesity than in lean women and circulating omentin levels are reduced [118,119]. Thus, expanding visceral fat and worsening blood glucose control diminish PVAT-derived omentin-1, fostering a pro-inflammatory, oxidative adipose environment that accelerates endothelial damage and vascular injury.

Evidence supports direct sex-dependent PVAT-resistin signaling in hypertensive rat models, where male mPVAT exhibited approximately a two-fold higher resistin expression than female PVAT [120]. Male-derived PVAT hampers ATP-sensitive potassium (K_ATP_) channel–mediated vasorelaxation, and exogenous resistin can mimic these effects, supporting a local role for resistin in sex-dependent PVAT regulation [121]. Separately, diabetic/obese mouse models demonstrate increased resistin expression in aortic PVAT and a causal contribution of PVAT-derived resistin to vascular AP-1 signaling [78]. However, whether PVAT-derived resistin is differentially regulated between sexes during prediabetes or diabetes remains unknown.

Sex differences in leptin are well established systemically, with women exhibiting higher circulating leptin concentrations than men even after accounting for total fat mass [122]. However, circulating leptin does not directly reflect PVAT-derived leptin and may be influenced by fat distribution and hormonal status. In T2DM, leptin resistance may promote PVAT inflammation and oxidative stress, shifting PVAT depot function toward a pro-contractile and pro-atherogenic phenotype [68,123]. The protective function of leptin in females tends to diminish in diabetes, leading to reduced vasorelaxation and increased vascular stiffness. Although leptin-mediated vascular effects may differ by sex and become altered in diabetes, evidence directly linking these differences to PVAT-derived leptin remains limited [32,123]. Thus, sex-dependent leptin signaling may contribute to differences in vascular dysfunction; however, these effects cannot yet be attributed specifically to PVAT-derived leptin [123].

Evidence suggests sex-dependent PVAT–apelin signaling in metabolic dysfunction. In female stroke-prone spontaneously hypertensive Zucker fatty (SHRSPZF) rats with metabolic syndrome, superior mPVAT enhanced vasorelaxation, and higher PVAT apelin expression was associated with this protective effect [124]. In a separate model, prediabetic female hereditary hypertriglyceridemic (HTG) rats showed preserved thoracic aortic PVAT function involving compensatory NO/H_2_S signaling, although apelin was not measured [73]. Human evidence is predominantly systemic: in the 9-year DESIR cohort, baseline plasma apelin was similar between sexes, but lower levels predicted incident T2DM, with favorable metabolic associations observed mainly in men [125]. Conversely, another prospective cohort associated higher plasma apelin with incident T2DM specifically in men, highlighting inconsistency in circulating apelin findings [126]. Thus, while experimental evidence suggests a potential sex-dependent role for PVAT–apelin signaling, its role in human progression from prediabetes to diabetes remains unclear.

Metabolic stress can remodel PVAT inflammatory signaling, but direct male-versus-female cytokine comparisons remain limited and vary by model and depot. Available studies suggest that male PVAT may exhibit greater inflammatory or oxidative remodeling in some settings, whereas female protection can diminish with aging, ovarian-hormone loss, or advanced diabetes [127,128,129,130]. Our prior studies showing greater vascular impairment in diabetic females than males are consistent with loss of female vascular protection during disease progression, but they should not be interpreted as direct evidence of PVAT-derived cytokine mechanisms [3,131,132,133,134].

Together, these findings suggest that current evidence is insufficient to determine when the sex-specific differences in PVAT function first emerge, and further studies are required to establish whether these differences vary with cardiometabolic status.

### 6.2. Other Vascular-Associated Cardiometabolic Diseases

A key experimental question is whether PVAT itself transfers a sex-dependent vascular phenotype. Small et al. addressed this in stroke-prone spontaneously hypertensive rats by studying mesenteric arteries with PVAT and by transferring male PVAT to female vessels [120]. Female arteries showed greater cromakalim-induced relaxation, and exposure to male PVAT attenuated this response [120]. In addition, male PVAT expressed approximately twofold more resistin, identifying a candidate mediator; however, the study was performed in a specific hypertensive rat model and cannot be generalized to all vascular beds or metabolic diseases [120]. Resistin-related TLR4 and NF-κB signaling provides a plausible pathway, although this pathway was not demonstrated specifically in PVAT, and additional loss-of-function studies are needed to establish causality [135].

Sex-dependent metabolic-vascular crosstalk is particularly relevant to the development of atherosclerosis. The sexual dimorphism of cholesteryl ester transfer protein (CETP) affects PVAT differently, though CETP is a well-known protein that increases the risk of atherosclerosis by lowering high-density lipoprotein cholesterol (HDL-C) levels [136]. In male mice, increased CETP expression reduced PVAT-derived NO release and increased NADPH oxidase 1/2 (NOX1/2)-dependent ROS, thereby impairing anti-contractile function, and PVAT function was restored by ex vivo estrogen treatment [136]. In contrast, CETP expression in female mice increased phospho-eNOS (p-eNOS) levels, facilitating NO production and thereby maintaining anti-contractile and anti-inflammatory properties and supporting sex-dependent regulation of PVAT vascular function [136]. These findings demonstrate sex-dependent PVAT responses in this experimental model but should not be generalized across vascular beds or atherosclerotic conditions; they also support a potential contribution of estrogen to female PVAT protection, which may be modified by menopause and aging [130,137].

Human evidence remains largely associative. In a human cross-sectional study, postmenopausal women showed increased cardiac fat and an elevated risk of coronary heart disease, although cardiac fat does not necessarily correspond to the specific PVAT depots examined experimentally [138]. In addition, one study comparing women with men, all with a mean age of 65+ years, has demonstrated that higher perivascular inflammation is associated with a higher prevalence of thin-cap fibroatheroma and macrophage accumulation, predominantly in women [139]. However, these observational findings do not establish causality. Overall, sex may modify PVAT-associated inflammation and atherosclerosis, but the underlying mechanisms remain context-dependent.

Obesity further demonstrates how metabolic stress leads to sex-specific PVAT remodeling. Ivatt et al. showed in an HFD-induced obesity model that mPVAT from obese male mice selectively impaired endothelium-dependent vasorelaxation, while females retained their PVAT-mediated vasorelaxation [140]. According to RNA sequencing analysis, the impairment is due to mPVAT from obese males having increased inflammatory gene expression, including nitric oxide synthase 2 (NOS2) and CD68, compared to the obese female group [140]. However, protection of female PVAT is not uniform across obesity models. Victorio et al. showed that obesity-induced PVAT dysfunction appeared earlier in female than in male mice, with the phenotype varying according to diet composition and duration [141]. These apparently divergent findings suggest that sex alone does not determine PVAT susceptibility; rather, diet, the duration and severity of metabolic stress, and the functional endpoint examined may influence the direction and magnitude of sex-dependent PVAT dysfunction. Accordingly, current evidence does not support a universal designation of female PVAT as either protected from or more susceptible to metabolic dysfunction.

Overall, these studies indicate that sex differences in PVAT remodeling are context dependent, with their presence, direction, and magnitude varying by vascular bed, disease model, and metabolic stage. Further direct and longitudinal evidence is needed to establish whether these mechanisms can be targeted to improve vascular outcomes during progression from prediabetes to diabetes.

## 7. Sex-Specific PVAT Involvement in Therapeutic Outcomes

Therapeutic strategies relevant to PVAT should be interpreted according to whether PVAT modulation has been directly demonstrated or inferred from broader systemic effects. Several preclinical interventions provide relatively direct evidence of PVAT modulation. Calycosin restored the anticontractile activity of thoracic PVAT in obese mice while increasing PVAT adiponectin, activating AMPK/eNOS signaling, and reducing TNF-α and superoxide [142]. Importantly, similar effects were reproduced with ex vivo treatment, supporting a local PVAT mechanism. Methotrexate also modified PVAT inflammatory and adiponectin signaling through an AMPK-dependent pathway, while PVAT-conditioned-medium experiments linked these changes to improved endothelial vasodilation [96].

Systemic interventions can also improve PVAT phenotype, although such findings should not be interpreted as evidence that PVAT is their primary therapeutic target. Exercise reduces PVAT inflammation and oxidative stress in parallel with improved endothelial function in diabetic mice, and moderate-intensity exercise attenuates obesity-associated PVAT alterations in female mice [143,144]. Dietary supplementation with chia oil similarly reduces PVAT inflammation and endothelial dysfunction in obese male mice [145]. Omentin-1 supplementation restored PVAT-mediated anticontractile activity and reduced superoxide production in PVAT-containing vessels in a diabetic rat model, providing more direct evidence of improved local PVAT-vascular function [64]. In contrast, adiponectin mimetics such as ALY688 improve systemic glucose and lipid metabolism, but their specific actions on PVAT require direct evaluation [146]. Thus, interventions that directly demonstrate PVAT-dependent vascular effects should be distinguished from systemic therapies that may secondarily modify PVAT. Since PVAT composition varies widely across vascular beds, and different types of adipose tissue have distinct functions, therapeutic responses may also vary across PVAT depots. Therefore, isolating PVAT from a single depot, such as aortic PVAT, might not accurately reflect PVAT characteristics in coronary, mesenteric, or resistance vessels [9,13,128,147]. This heterogeneity also has therapeutic implications, as an intervention targeting aortic PVAT may not produce the same molecular or functional effects in coronary or mPVAT.

The therapeutic implications of sex-specific PVAT biology remain largely hypothetical. Sex differences in therapeutic responses or adverse effects have been reported for several cardiometabolic drug classes, including glucagon-like peptide-1 (GLP-1) receptor agonists, but most of these data derive from systemic pharmacology rather than studies designed to determine whether PVAT mediates the observed sex difference [148,149]. Apparent sex differences may also be influenced by dose, body composition, age, menopausal status, treatment discontinuation, and unequal enrollment. Therefore, claims of sex-specific therapeutic benefit should be supported by appropriately designed sex-stratified analyses or robust meta-analyses reporting effect sizes. At present, no clinical treatment guideline targets PVAT or recommends therapy according to a validated sex-specific PVAT phenotype. Estrogen/ER signaling provides a relatively strong mechanistic rationale for potential sex-dependent modulation of PVAT, although systemic estrogen replacement is not a PVAT-specific intervention. Downstream protective pathways, including adiponectin signaling, as well as inflammatory and redox pathways, may represent potential therapeutic targets; however, their PVAT-specific and sex-dependent therapeutic effects remain insufficiently established. These approaches therefore remain experimental and require direct validation in sex-stratified, PVAT-focused studies.

Overall, among candidate pathways, restoration of adiponectin–AdipoR signaling is mechanistically attractive because of its established anticontractile, anti-inflammatory, and metabolic actions and evidence for sex-dependent vascular responsiveness. Future therapeutic development should therefore prioritize pathways directly demonstrated within a defined PVAT depot to causally influence vascular function in a sex-dependent manner.

## 8. Limitations and Future Directions in Sex-Specific PVAT Biology

The evidence base should be interpreted at three levels. Direct evidence comes mainly from animal vascular preparations in which PVAT is retained, removed, transferred, or pharmacologically manipulated; these studies support local effects on vascular tone and selected metabolic signals. In humans, direct sex-specific evidence would require demonstrating differences between women and men in PVAT-derived mediators or PVAT–vascular interactions, ideally supported by functional or ex vivo manipulation of PVAT or its signaling pathways. Although human studies have examined these PVAT-related endpoints, sex-stratified mechanistic evidence remains scarce. Indirect evidence includes circulating adipokines, systemic hormone studies, and vascular experiments in which PVAT was not manipulated. Speculative conclusions include extrapolation from these findings to human prediabetes progression or sex-specific treatment response. Most mechanistic evidence remains preclinical, and changes in PVAT during the transition from normoglycemia to prediabetes and then overt diabetes are not yet well characterized.

Another critical gap is the limited characterization of sex-specific inflammatory and immune mechanisms within PVAT. Sex differences in PVAT morphology and its modulatory effects on endothelial function and vascular tone have been reported, but the specific cytokines, immune cell populations, and inflammatory pathways that drive male versus female PVAT remodeling remain poorly defined [128,150]. This gap is particularly important because adipose tissue inflammation, oxidative stress, and impaired NO bioavailability are central mechanisms linking metabolic dysfunction with endothelial impairment and vascular disease [151,152].

Future studies should use adequately powered and sex-balanced designs. Sample-size calculations should be based on a prespecified primary PVAT-dependent endpoint and powered to detect sex-by-disease or sex-by-treatment interactions. The required sample size should be determined a priori from the expected effect size, variability, experimental model, vascular bed, statistical design, and desired power. Human studies designed to detect sex-specific PVAT effects will generally require sufficiently large cohorts to account for relevant biological and clinical variability, including age, adiposity, reproductive or menopausal status, hormone exposure, medications, and disease severity. Because no validated threshold currently defines a clinically meaningful sex-specific PVAT effect, studies should prespecify relevant vascular or PVAT endpoints and report effect sizes with confidence intervals. They should also report age, estrous-cycle stage, menopausal status, hormone therapy, and circulating sex-hormone concentrations. Animal studies should incorporate gonadectomy and hormone-replacement controls where appropriate and match male and female groups for adiposity, glycemic severity, disease duration, and vascular injury. Because PVAT heterogeneity varies across vascular beds, in addition to sex-specific studies, extensive vessel-specific studies are needed to determine how PVAT-specific mechanisms contribute to each vessel. Causal experiments should compare vessels with and without PVAT and use conditioned media, transplantation, cross-sex transfer, or cell-specific perturbation to distinguish PVAT-mediated effects from systemic vascular differences.

The contribution of sex hormones to PVAT-mediated vascular regulation remains insufficiently defined. Studies should distinguish ERα- and ERβ-dependent actions within adipocytes, immune cells, endothelium, and VSMCs and should test how receptor signaling changes with reproductive aging and metabolic disease [153,154]. Progesterone and androgen pathways require similarly cell-specific and vascular-bed-specific investigation rather than inference from systemic hormone concentrations [100,155].

Moreover, comprehensive profiling of the sex-specific PVAT secretome remains limited. Future studies should combine single-cell or single-nucleus RNA sequencing, spatial transcriptomics, proteomics, lipidomics, extracellular-vesicle analysis, immune profiling, mitochondrial assays, and vascular reactivity. These approaches should distinguish adipocyte-derived signals from stromal, endothelial, and immune-cell sources and validate candidate secreted proteins in vessel-specific human PVAT. A feasible approach to obtain more direct human evidence would be to collect paired PVAT and vascular tissue from women and men undergoing cardiovascular surgery, with careful characterization of metabolic status, age, reproductive or menopausal status, and hormone exposure. Ex vivo vascular functional studies could then be combined with measurement of PVAT-derived mediators and targeted manipulation of candidate pathways. Prospective sex-stratified studies could further determine whether these PVAT phenotypes are associated with subsequent vascular or metabolic disease progression.

Lastly, an additional emerging area is the potential gut microbiome–PVAT axis. Experimental data demonstrate crosstalk between the microbiota and PVAT, including evidence that intestinal microbiota promoted B2-cell recruitment and activation in PVAT, whereas microbiota depletion reduced PVAT B2-cell activation and atherosclerosis in a mouse model [156]. Thus, gut microbial composition and microbial metabolites can influence systemic metabolism, inflammatory tone, and adipose-tissue biology, raising the possibility that microbiome-dependent signals also contribute to PVAT remodeling [7,157]. Because both gut microbial composition and cardiometabolic responses can differ by sex, this pathway may also contribute to sex-dependent PVAT phenotypes [7,157]. However, direct evidence linking sex-specific alterations in the gut microbiome to PVAT-mediated vascular dysfunction remains limited, and this represents an important area for future investigation.

## 9. Conclusions

PVAT is a metabolically active component of the vascular wall whose phenotype can shift from anticontractile and metabolically supportive to inflammatory, oxidative, and pro-contractile during cardiometabolic disease. Although evidence for sex-dependent PVAT dysfunction is compelling in selected animal models, findings remain inconsistent across vascular beds and are limited in humans, particularly during the transition from prediabetes to overt diabetes. Rather than assuming uniform male or female protection, future studies should use sex-balanced designs and direct PVAT manipulation to define causal, vessel-specific mechanisms. From a translational perspective, identifying such mechanisms may reveal opportunities for sex-informed therapeutic intervention; however, current pharmacological, lifestyle, and hormone-based approaches exert largely systemic effects and have not been shown to selectively correct sex-specific PVAT abnormalities. Establishing PVAT as a therapeutic target will therefore require evidence that modifying a defined PVAT pathway produces vascular benefit in a sex-dependent manner.

## Figures and Tables

**Table 1:** Comparison of PVAT with other adipose tissues [18].

Feature	White Adipose Tissue (WAT)	Brown Adipose Tissue (BAT)	Beige Adipose Tissue	Perivascular Adipose Tissue (PVAT)
Primary function	Energy storage	Heat production	Inducible thermogenesis	Regulation of vascular homeostasis and vascular tone
Anatomical location	SAT and visceral depots	Interscapular/cervical regions	Scattered within WAT depots	Surrounds blood vessels
Cell morphology	Large unilocular lipid droplet	Small multilocular lipid droplets	Intermediate phenotype	White-, brown-, or beige-like phenotype depending on vascular bed
Mitochondrial abundance	Low	Very high	Moderate-high	Variable and vascular-bed specific
UCP1 expression	Minimal	High	Inducible	Thoracic PVAT often BAT-like; abdominal PVAT more WAT-like
Main secretory products	Leptin, adiponectin, resistin	Fibroblast growth factor 21 (FGF21) and thermogenic mediators	Thermogenic cytokines	Adiponectin, leptin, omentin, cytokines, NO, hydrogen sulfide (H_2_S), angiotensin II (Ang II)
Thermogenic capacity	Very low	High	Adaptive/intermediate	Variable and depot-specific
Immune-cell composition	Macrophages and lymphocytes	Lower inflammatory profile	Variable	Rich immune-cell population with vascular interactions
Role in vascular regulation	Indirect	Minimal	Limited	Direct regulation through anticontractile and contractile factors
Vascular bed specificity	Not inherently vascular bed specific; varies by anatomical depots	Not inherently vascular-bed-specific	Not inherently vascular-bed-specific	Highly vascular-bed-specific; Phenotype and function vary by vascular location

## Data Availability

No new data were created or analyzed in this study. Data sharing is not applicable.

## Abbreviations

The following abbreviations are used in this manuscript:

ACh	Acetylcholine
AdipoR1	Adiponectin receptor 1
AdipoR2	Adiponectin receptor 2
ADRF	Adipocyte-derived relaxing factor
Akt	Protein kinase B
AMPK	Adenosine monophosphate-activated protein kinase
Ang	Angiotensin
AP-1	Activator protein 1
ApoE	Apolipoprotein E
AT1	Angiotensin II type 1 receptor
BAT	Brown adipose tissue
BMI	Body mass index
BK_Ca_	Large-conductance calcium-activated potassium
CAP1	Adenylyl cyclase-associated protein 1
CCL2	C-C motif chemokine ligand 2
CCL5	C-C motif chemokine ligand 5
CD34^+^	Cluster of differentiation 34
CD4^+^	Cluster of differentiation 4
CDC	Centers for Disease Control
CETP	Cholesteryl ester transfer protein
DOCA	Deoxycorticosterone acetate
eNOS	Endothelial nitric oxide synthase
ERs	Estrogen receptors
ERK	Extracellular signal-regulated kinase
ERα	Estrogen receptor alpha
ERβ	Estrogen receptor beta
F/G	Filamentous-to-globular actin ratio
FGF21	Fibroblast growth factor 21
GLP1	Glucagon like peptide 1
HFD	High-fat diet
HIF1α	Hypoxia-inducible factor-1 alpha
H_2_O_2_	Hydrogen peroxide
H_2_S	Hydrogen sulfide
HOMA-IR	Homeostatic model assessment of insulin resistance
IDF	International Diabetes Federation
IFN-γ	Interferon-γ
IL-6	Interleukin-6
IL-8	Interleukin-8
IR	Insulin resistance
JAK	Janus kinase
JAK2	Janus kinase 2
K_ATP_	ATP-sensitive potassium channel
KCa	Calcium-activated potassium channel
M1	Classically activated macrophage
M2	Alternatively activated macrophage
MAPK	Mitogen-activated protein kinase
MCP-1	Monocyte chemoattractant protein-1
mPVAT	Mesenteric perivascular adipose tissue
NADPH	Nicotinamide adenine dinucleotide phosphate
NF-κB	Nuclear factor kappa B
NLRP3	NOD-like receptor family, pyrin domain containing 3
NO	Nitric oxide
NOX1	NADPH oxidase 1
NOX2	NADPH oxidase 2
NOX4	NADPH oxidase 4
Nrf2	Nuclear factor erythroid 2-related factor 2
OPN	Osteopontin
PI3K	Phosphoinositide 3-kinase
PKG	Protein kinase G
PPAR-α	Peroxisome proliferator-activated receptor alpha
PPAR-δ	Peroxisome proliferator-activated receptor delta
PVAT	Perivascular adipose tissue
PVCFs	PVAT-derived contracting factors
PVRFs	PVAT-derived relaxing factors
RAAS	Renin–angiotensin–aldosterone system
RNA-seq	RNA sequencing
ROS	Reactive oxygen species
SAT	Subcutaneous adipose tissue
SHR	Spontaneously hypertensive rat
STAT	Signal transducer and activator of transcription
T2DM	Type 2 diabetes mellitus
TF	Therapeutic fasting
TGF- β	Transforming Growth Factor-β
TLR4	Toll-like receptor 4
TNF-α	Tumor necrosis factor alpha
TXA_2_	Thromboxane A_2_
UCP1	Uncoupling protein 1
VAT	Visceral adipose tissue
VEGF	Vascular endothelial growth factor
VSMC	Vascular smooth muscle cell
WAT	White adipose tissue
